# Health Tract, No. 4

**Published:** 1860-02

**Authors:** 


### HEALTH TRACT, No. 4.

### NINE NEVERS.

Never write a letter or a line in a passion.

Never spit or blow your nose on the sidewalk.

Never find a fault until you are as sure as you are of your existence that a fault has been committed.

Never say what you would do under any given circumstances.

Never disparage another by name in a letter.

Never get in a rage.

Never utter a syllable in a passion.

Never refuse to pay a debt when you have the money in your pocket.

Never take physic until you have tried patience.

### CAUSES OF DISEASE.

The complaints of people are in a measure innumerable; every now and then a peculiarity of ailment is presented which is not recorded in any book extant; just as new questions of law are constantly arising. But while the effects of disease are so numerous, the causes of them may be reduced down so low as to be all told in the number five:

*First*—Poisons.

*Second*—Improper eating.

*Third*—Variations of atmosphere.

*Fourth*—Occupations.

*Fifth*—Hereditary tendencies; which last, indeed, is a modification of the first.

Of the four, by far the most frequent causes of disease are found in the food we eat, and in the air we breathe, the rectification of both of which is within our own power; requiring only a moderate amount of intelligence, but a large share of moral power, that is, a resolute self-denial. It thus follows, that death, short of old age, is chargeable to man himself; that in an important sense, the great mass of those who die short of threescore years and ten, are the authors of their own destruction. And each should inquire, "To what extent am I chargeable with my own ailments?"

### BURYING ALIVE.

"Tis well," were the last recorded words of the great Washington, uttered in reference to his burial.

"Do not let my body be put into the vault in less than three days after I am dead," and, looking earnestly into his secretary's face, he continued, "Do you understand me?" "Yes," said Mr. Lear. "'Tis well," replied Washington, and spoke no more.

The great Dr. Physic left an injunction that a blood-vessel should be severed before he was buried, in order to make it certain that he was dead.

The marvellous stories put in circulation by the credulous, in reference to the turning of bodies, and the tearing of the grave-clothes in the fearful struggle for breath, are without any rational foundation. If a hot iron raises no blister on the skin, or if a severed artery does not bleed, there can be no reasonable ground for doubting that death has taken place. These tests should be applied not sooner than eight or ten hours after the apparent decease.

